# Cultivable oral bacteriota dysbiosis in mechanically ventilated COVID-19 patients

**DOI:** 10.3389/fmicb.2022.1013559

**Published:** 2022-10-28

**Authors:** Iwona Gregorczyk-Maga, Mateusz Fiema, Michal Kania, Jolanta Kędzierska, Estera Jachowicz, Dorota Romaniszyn, Jadwiga Wójkowska-Mach

**Affiliations:** ^1^Faculty of Medicine, Institute of Dentistry, Jagiellonian University Medical College, Krakow, Poland; ^2^Department of Endocrinology, University Hospital, Krakow, Poland; ^3^Doctoral School of Medicine and Health Sciences, Jagiellonian University Medical College, Krakow, Poland; ^4^Chair of Metabolic Diseases, Faculty of Medicine, Jagiellonian University Medical College, Krakow, Poland; ^5^Microbiology Unit, University Hospital, Krakow, Poland; ^6^Chair of Microbiology, Faculty of Medicine, Jagiellonian University Medical College, Krakow, Poland

**Keywords:** oral microbiota, dysbiosis, COVID-19, mechanical ventilation, ARDS

## Abstract

Potential interactions between the SARS-CoV-2 virus and the human oral microbiota are currently investigated widely. Patients with COVID-19 requiring mechanical ventilation in an intensive care unit (ICU) setting are at high risk of developing severe complications, including ventilator-associated pneumonia, thus making oral health management important. The aim of this study was to evaluate the oral health status and assess the dysbiosis of cultivable oral bacteriota in COVID-19 patients hospitalized in an ICU with acute respiratory distress within 36 h following intubation. In this prospective cohort study, we recruited 56 adult COVID-19 patients that qualified for mechanical ventilation in the Temporary ICU for COVID-19 Patients of the University Hospital in Krakow. On admission to the ICU, oral health of patients was assessed using the modified Beck Oral Assessment Score (BOAS). Four oral habitats were sampled, namely the buccal mucosa, tongue, buccal dental surface and gingival pocket. Microorganisms were identified by MALDI/TOF mass spectrometry. The mean age of the study population was 66.5 ± 12.7 years, there were 24 (42.9%) females. All patients included in this study were intubated and ventilated in the ICU, with a corresponding high mortality rate (76.8%). On admission to ICU, 76.8% subjects scored 11–20 on the BOAS scale (median 12 [IQR 10–14]), indicating moderate or severe dysfunction of oral health. Potentially pathogenic bacteria were identified in the oral microbiota samples, including *Acinetobacter baumannii, Enterococcus faecalis, Escherichia coli and Klebsiella pneumoniae* in 23.2%, 39.3%, 17.9%, and 19.6% of patients, respectively. Lactobacillus spp. were present in 57.1% subjects. The mean CFU counts of all bacteria strains in dental brushes were 9.3E+5 (1.4E+6) and in gingival pockets 7.6E+5 (1.4E+6). The highest CFU counts were observed for *Enterococcus* spp. and, *Lactobacillus* spp., although these did not differ significantly from CFU counts of *Streptococcus* spp. and *Staphylococcus* spp. In this report we comprehensively characterized the oral health condition and cultivable oral bacteriota in COVID-19 patients hospitalized in an ICU with acute respiratory distress within 36 h following intubation. The oral bacteriota showed significant qualitative and quantitative dysbiosis. Hospitalization in an ICU and mechanical ventilation are important factors leading to oral dysbiosis in SARS-CoV-2 patients.

## Introduction

Coronavirus disease 2019 (COVID-19), caused by a severe acute respiratory syndrome coronavirus 2 (SARS-CoV-2), has caused a global pandemic and resulting serious public health crisis (Zhu et al., 2020; World Health Organization, 2022). While most COVID-19 patients have minor symptoms, ~15% of hospitalized patients require admission to an intensive care unit (ICU; Terlecki et al., 2021). These patients exhibit respiratory failure with a systemic inflammatory reaction and multiple-organ dysfunction, requiring oxygen supplementation and, in some cases mechanical ventilation (Weiss and Murdoch, 2020).

Over 1,000 bacterial species have been reported to reside in the oral cavity (Dewhirst et al., 2010). In healthy individuals the oral bacteriota is dominated by Actinobacteria (Acctinomyces, Corynebacterium and Rothia), Bacteroides (Capnocytophaga, Porphyromonas and Prevotella), Firmicutes (Granulicatella and Streptococcus), Fusobacteria, Proteobacteria (Haemophilus and Neisseria; Zaura et al., 2001). The oral cavity can be divided into several microbiologically distinct niches, including saliva, soft tissue surfaces of the oral mucosa and tongue, and hard tissue surfaces of teeth (Xu et al., 2015; Zhang et al., 2018). Of the most common species, 54% is cultivable and identifiable, 14% is cultivable but not easily identified, and 32% cannot be cultivated, remaining in dormant state (Caselli et al., 2020). Recent research of oral microbiota resulted in two large databases: Human Microbiome Project (HMP) and Human Oral Microbiome Database (HOMD). HMP contains microbiome data from 5 main environments: the oral cavity, nasal cavity, vagina, gut and skin. Data in HOMD encompasses oral microbiota composition (Li et al., 2022).

Previous studies of changes in microbiome, including the oral microbiota, showed a significant reduction of microbial diversity in SARS-CoV-2 affected patients (Iebba et al., 2021; Wu et al., 2021; Uehara et al., 2022). These changes comprised decreased abundance of Neisseria, Corynebacterium, Aggregatibacter, Treponema, and Pseudomonas genus, and *Prevotella intermedia* in the oral cavity of COVID-19 patients. Importantly, the loss of comensal Neisseria, such as *N. subflava* and *N. mucosa*, and *Prevotella* spp. acting as a local oral probiotic, can lead to severe imbalance in the oral microbiota composition (Weyand, 2017; Rafiqul Islam et al., 2022). Enrichment of Campylobacter, Granulicatella, Veillonella and Filifactor genus was also observed, that can be of clinical significance, as those are taxa associated with periodontitis (Wu et al., 2021). *Veilonella* spp. has been also reported to induce proinflammatory responses (Haran et al., 2021). Furthermore, increased abundance of opportunistic *Hemophilus parainfluenzae* in the oral cavity can predispose patients to respiratory tract infections (Iebba et al., 2021; Wu et al., 2021). Another report revealed that COVID-19 patients had a higher abundance of *Enterococcus* spp. in the oral cavity, linking respiratory pathogens with gut microbiome abnormalities (Rafiqul Islam et al., 2022). The dysbiosis was even more pronounced in severe course of infection and long-COVID-19, suggesting that its extent can be treated as an indicator of infection severity (Haran et al., 2021; Wu et al., 2021; Fiema et al., 2022; Rafiqul Islam et al., 2022). The use of antibiotics in COVID-19 patients was also associated with independent oral and gut microbiome profiles (Wu et al., 2021).

A high prevalence of oral health problems, such as xerostomia, mucosal blistering, mouth rash and lip necrosis has been observed in patients with COVID-19 (Aragoneses et al., 2021). Several trials have correlated poor oral hygiene with hyper-inflammation (Kamel et al., 2021), and poor oral health in patients with caries and periodontitis may play a significant role in the development of severe complications of COVID-19 in patients managed in the ICU (Hocková et al., 2021; Marouf et al., 2021). Moreover, during prolonged endotracheal intubation, dysbiotic oral microbiota can colonize the lower respiratory tract. These patients are at high risk for developing bacterial ventilator-associated pneumonia (VAP). The oral management of these patients in an ICU is critical as oral care has been shown to reduce the incidence of VAP (Bao et al., 2020; Luyt et al., 2020).

The aim of this study was to evaluate the oral health status and assess the dysbiosis of cultivable oral bacteriota in COVID-19 patients hospitalized in an ICU with acute respiratory distress in the early post-intubation period.

## Materials and methods

### Study design and participants

In this prospective cohort study, we recruited 56 consecutive adult COVID-19 patients that qualified for mechanical ventilation in the Temporary ICU for COVID-19 Patients of the University Hospital in Krakow (UH) between January 31st and September 1st 2021. University Hospital in Krakow coordinated the care for patients with SARS-CoV-2 infection in Lesser Poland and was responsible for the hospitalization of patients with COVID-19 requiring specialized treatment.

Patients were diagnosed with COVID-19 according to WHO and Polish guidelines with the use of RT-PCR (Diagnostic testing for SARS-CoV-2 [internet], 2022; Flisiak et al., 2022). The COVID-19 treatment algorithm in patients admitted to UH was based on constantly updated recommendations of the Polish Association of Epidemiologists and Infectiologists (Flisiak et al., 2022), including concurrent probiotic use in patients undergoing antibiotic therapy.

The inclusion criteria for this study were as follows: 1. SARS-CoV-2 infection confirmed by RT-PCR assay of nasal and pharyngeal swabs upon hospital admission, 2. Admission to the ICU, 3. Signed consent to participate in the study and 4. Intubation due to COVID-19 related pneumonia and acute respiratory distress syndrome (ARDS) within the preceding 36 h from commencement of the study procedures.

The patients included in the study were admitted from emergency wards (either at UH or non-UH) or transferred from another ward dedicated for COVID-19 patients (UH or outside UH).

Demographic and clinical data were gathered from the hospital electronic medical records. The database included information on age, sex, date of COVID-19 diagnosis (defined as the first positive result of antigen and PCR test from nasopharyngeal swab), date of admission to the hospital, institution of the patients’ origin (emergency ward, hospital ward), date of discharge or death, date of admission to the ICU, date of intubation, COVID-19 severity on WHO Clinical Progression Scale (Supplementary Table S1; Marshall et al., 2020), comorbidities [previous diagnosis of diabetes, arterial hypertension, heart failure (HF), history of MI or stroke, ischemic heart disease, atrial fibrillation (AF), chronic kidney disease (CKD), chronic obstructive pulmonary disease (COPD)] and pre-intubation treatment [remdesivir, antibiotic, days of antibiotic treatment (DOT) before intubation (the number of days a patient receives an antibiotic independent of dose), proton pump inhibitor]. CVD and cardiovascular risk factors were identified based on a medical history of prehospital diagnosis or treatment. Other chronic comorbidities were also diagnosed based on earlier clinical notes available in the medical records. Baseline laboratory results [C-reactive protein (CRP), procalcytonin (PCT), interleukin-6 (IL-6), D-dimer, white blood count (WBC), creatinine] were also extracted.

### Oral health assessment

On admission to the ICU, oral health was assessed using a modified BOAS, consisting of five subscales, namely assessment of lips, mucosa and gingiva, tongue, teeth, and saliva. A higher score reflects dysfunction or tissue injury. BOAS scores range from 5 (no oral dysfunction) to 20 (severe dysfunction), and a score >5 is abnormal (Beck, 1979; Ames et al., 2011; Supplementary Table S2).

### Oral cavity sampling methods

Four oral habitats were sampled by a trained dentist: the buccal mucosa, the tongue, buccal dental surface and gingival pocket, with the latter two only in patients with dentition. Specimens from the posterior dorsum of the tongue and buccal mucosa were collected using ESwab™ (Demuyser et al., 2018), which combines a COPAN-invented flocked swab with 1 ml of Liquid Amies in a plastic, screw cap tube. Dental plaque was collected from buccal dental surface side using Tooth Cleanic KerrHawe-KWX-OP-SZ-011, and after collection the brush was placed in 1 ml of Liquid Amies in a plastic screw cap tube. PerioPaper Strips (*n* = 3; Guentsch et al., 2011), designed to absorb or carry 0–1.2 μl of fluid, were used to collect gingival crevicular fluid (GCF) samples. The strips were placed in the gingival pocket for 30–45 s till its surface moistened. To minimize the risk of pre-analytical errors during sample collection, sterile gauze was used to remove excess saliva from the mucosa and dry the dental surfaces, preventing salivary contamination of GCF.

### Microbiological cultures

The samples were immediately delivered to the microbiological laboratory, where they were inoculated by the dilution method (dilutions −1 to −6) or qualitative culture method (swabs only) on the following media: McConkey (Graso, Biotech), Columbia (Lab-Agar, Biomaxima), Scheadler (Scheadler-Agra, Biomaxima), Bile Esculine Azide (Lab-Agar, Biomaxima), MRS Agar (Oxoid), Sabouraud Agar (Biomaxima). Media were aerobically incubated at 37°C (McConkey, Columbia, Bile Esculine Azide) or anaerobically at 37°C (M.R.S and Scheadler, GENbag Atmosphere Generators [BioMérieux, France] for 48 h). After incubation, the phenotypical colonies were counted and reported, and results were presented as CFU/ml (colony forming unit). After isolation, the microorganisms were identified by MALDI TOF mass spectrometry (Vitek MS Home bioMérieux).

Multiple analyses were performed to identify factors associated with oral health status, biodiversity and composition of oral commensal and potentially pathogenic bacterial microbiota, and in-hospital mortality.

### Ethics statement

The study and its protocol were approved by the Jagiellonian University Bioethics Committee, decision number 1072.6120.333.2020; December 7, 2020. Written informed consent was obtained from each subject prior to participation.

### Statistical analysis

PS Imago Pro v.6.0 and Statistica v.13 were used for all statistical analysis. The normality of continuous variable distribution was assessed using the Shapiro–Wilk test. Differences between groups were analyzed with Student’s *t*-test or nonparametric tests (Mann–Whitney *U*-test, Kruskal–Wallis ANOVA) when appropriate. Paired data were analyzed using the Wilcoxon test or Friedman’s ANOVA along with appropriate post-hoc tests. Continuous variables were presented as arithmetic means (x̄) ± standard deviations (SD) or as the median with interquartile range (IQR) when the data were not normally distributed. The distribution of categorical variables was described as counts and percentages. Statistical testing was completed to compare categorical variables using an independent sample Chi-squared test or Fisher’s exact test when appropriate, and dependent samples with McNemar’s test and Cochran’s Q ANOVA. A *p*-value of <0.05 was considered statistically significant. The Bonferroni correction was used for multiple comparisons.

## Results

### Demographic data and background

The study population included 56 patients admitted to an ICU ward with ARDS due to COVID-19 related pneumonia. The mean age was 66.5 ± 12.7 years, there were 24 (42.9%) females. The subjects for whom data was available were classified as obese (mean BMI 31.9 ± 5.8, data available for 35 subjects). The most prevalent comorbidities were hypertension (46.4%), diabetes (35.7%) and coronary artery disease (28.6%).

**Figure 1:**
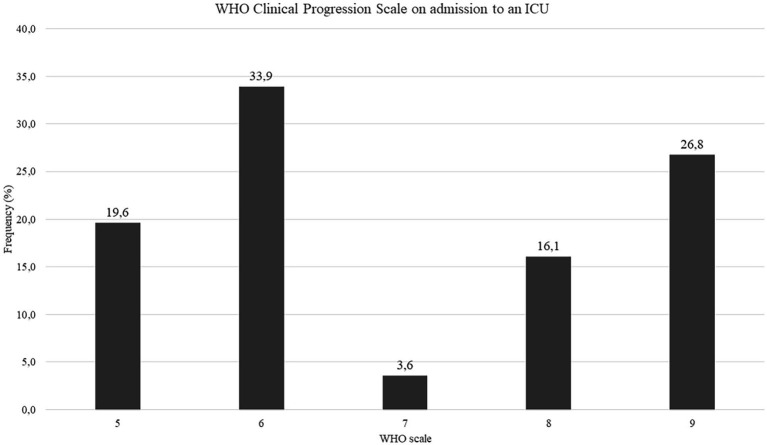
WHO Clinical Progression Scale on admission to an ICU, *N* = 56, data presented as percentages.

The median WHO Clinical Progression Scale score was 6 on admission to ICU, meaning patients required oxygen by NIV or high flow (Figure 1). Of the enrolled patients, 16 (28.6%) were transferred directly from the emergency department, 30 (53.6%) were transferred from another UH ward, and 10 (17.9%) were transferred from another ward outside UH. The mean time between admission to UH and intubation was 4.91 ± 5.56 days.

On admission to the hospital, inflammatory markers were increased, indicating a severe response to COVID-19 infection (Table 1). Systemic steroid therapy was used in 40 (76.9%) and antibiotics in 33 (63.5%, of whom 32 were treated with β-Lactam antibiotics) patients before admission to an ICU. Median antibiotic DOT before intubation was 8 (range 4–13 days).

**Table 1:** Baseline characteristics of study participants and outcomes of hospitalization.

Characteristics	Data available [*N*]	Value
Age [years]	56	66.5 (12.7)
Female [*n* (%)]	56	24 (42.9%)
BMI [kg/m^2^]	35	31.9 (5.8)
WHO Clinical Progression Scale on admission to an ICU	56	6 (6–9)
Source of admission [*n* (%)]	56	
Emergency ward		16 (28.6%)
Hospital ward		40 (71.4%)
Time from COVID-19 diagnosis^a^ to intubation [days]	53	6.95 (6.62)
Time from hospital admission to intubation [days]	53	4.91 (5.56)
Baseline BOAS, sum score^b^	49	12 (10–14)
Baseline BOAS, lips	56	2 (2–2)
Baseline BOAS, gingival and oral mucosa^b^	49	2 (2–3)
Baseline BOAS, tongue	56	2 (2–3)
Baseline BOAS, teeth^b^	49	3 (2–4)
Baseline BOAS, saliva	56	3 (2–3)
In-hospital death [*n* (%)]	56	43 (76.8%)
Laboratory findings		
CRP, first recorded ([mg/L], normal <5^c^)	55	158 (98.9)
PCT, first recorded ([ng/ ml], normal <0.5^c^)	55	5.1 (19.0)
IL-6, first recorded ([pg/ml], normal <7^c^)	54	141.2 (233.0)
WBC, first recorded ([10^3^/mm^3^], normal 4 × 10^3^–10×10^3c^)	54	10.8 (7.5)
Comorbidities [*n* (%)]		
COPD	56	3 (5.4%)
Smoking	52	7 (13.5%)
Diabetes	56	20 (35.7%)
History of neoplasm	56	15 (26.8%)
Hypertension	56	26 (46.4%)
Coronary artery disease	56	16 (28.6%)
Heart failure	56	6 (10.7%)
CKD	56	4 (7.1%)
In-hospital pharmacotherapy before intubation [*n* (%)]	52	
Steroid therapy		40 (76.9%)
Remdesivir		15 (29.4%)
Tocilizumab		11 (21.2%)
Antibiotic		33 (63.5%)
DOT before intubation [days]^d^		9.8 (10)
PPI		24 (46.2%)

data are presented mean (SD), median (Q1-Q3) or *N* [%]; BMI, body mass index; WHO, World Health Organization; ICU, intensive care unit; UH, University Hospital Cracow, Poland; CRP, C-reactive protein; PCT, procalcitonin; IL-6, interleukin 6; WBC, white blood cell count; COPD, chronic obstructive pulmonary disease; CKD, chronic kidney disease; PPI, proton pump inhibitor; BOAS, Beck Oral Assessment Scale; DOT, days of antibiotic therapy.

a Defined as first positive SARS-CoV-2 nasopharyngeal swab.

b Data for patients with dentition.

c According to local laboratory standards.

d Data for 33 patients undergoing antibiotic therapy.

### Clinical outcomes

All patients were intubated and ventilated in the ICU, and a corresponding high mortality rate was observed in the recruited patients (76.8%). There were no significant differences between survivors and non-survivors with regards to demographic characteristics, laboratory findings and oral health status (Supplementary Table S3).

Furthermore, there were no clinically relevant differences in the demographic characteristics, laboratory findings and mortality between the patients with different severities of COVID-19 on the WHO Progression Scale, the ward preceding admission to ICU, or previous steroid or antibiotic treatment.

### Oral health assessment

**Figure 2:**
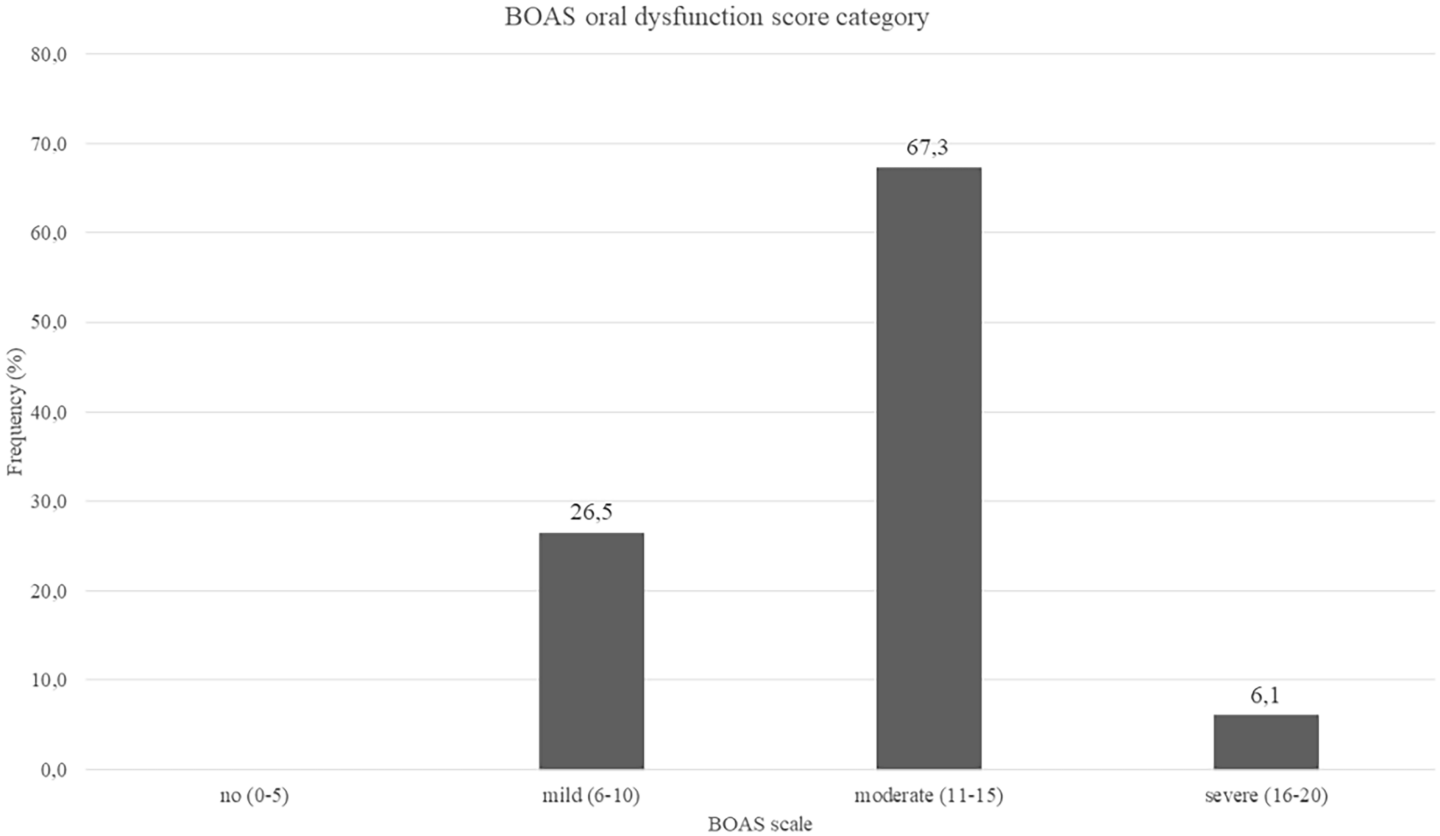
Baseline BOAS oral dysfunction score category, *N* = 49, data presented as percentages.

On admission to ICU, the median BOAS was 12 (IQR 10–14), and we found 76.8% subjects scored 11–20, indicating moderate or severe dysfunction of oral health (Figure 2). Furthermore, the BOAS score differed significantly between the subcategories (*p* < 0.001). Our data indicated teeth had a significantly higher BOAS score than lips and gingival/oral mucosa (*p* = 0.003, *p* = 0.011). Comparison of subjects with no or mild vs. moderate or severe dysfunction in BOAS score revealed that the latter were older (60, IQR 48–68 vs. 69.5, IQR 63–75, *p* = 0.006), had higher initial inflammatory markers (PCT 0.17, IQR 0.08–0.46 vs. 0.42, IQR 0.17–1.16, *p* = 0.034) and higher HbA1c% (5.9, IQR 5.3–6.35 vs. 6.75 IQR 6.15–8.28, *p* = 0.029). There was also a trend toward higher WHO Progression Scale score in patients with moderate or severe dysfunction as indicated by their BOAS score, but it did not reach statistical significance. Finally, there was a significant but weak positive correlation between the selected BOAS subscales and the time from COVID-19 infection detection.

### Bacteriological findings

**Figure 3:**
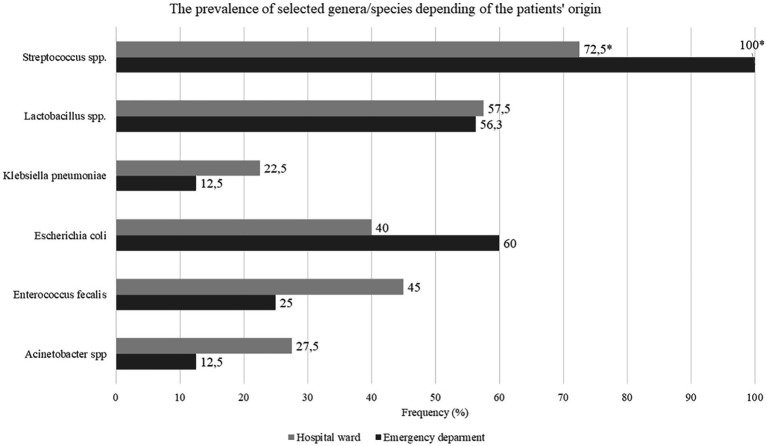
Number of patients with selected genera/species depending of the patients’s origin, *N* = 56, data presented as percentage.

In total, 32 genera and 70 bacterial species were identified in the study subjects (Table 2, full list in Supplementary Table S4). A number of strains were identified on the genera level [*Lactobacillus acidophilus/gasseri* (39 strains), *Streptococcus mitis/oralis* (100 strains), *Streptococcus salivarius ssp thermophilus/Str.salivarius ssp salivarius/Str. Vestibularis* (38 strains), *Lactobacillus casei/paracasei/rhamosus* (26 strains)]. Furthermore, multiple, potentially pathogenic bacteria were identified in the oral microbiota samples, including *Acinetobacter baumannii, Enterococcus faecalis, Escherichia coli* and *Klebsiella pneumoniae* in 23.2%, 39.3%, 17.9%, and 19.6% of patients, respectively (Figure 3). *Lactobacillus* spp. was present in 57.1% of patients, and Cariogenic *S. mutans* was identified in one subject.

**Table 2:** Baseline qualitative microbiological characteristics of study participants.

Characteristics	Data available [*N*]	Value	*p*
Total number of genera	56	32	–
Total number of species	56	70	–
Number of genera, all sites^a^	56	5 [4–6]	NS
Number of species, all sites^a^	56	6 [5–8]	NS
Number of patients with selected genera/species	56		
Acinetobacter baumannii		13 [23.2%]	–
Enterococcus faecalis		22 [39.3%]	–
Escherichia coli		10 [17.9%]	–
Klebsiella pneumoniae		11 [19.6%]	–
Lactobacillus spp.		32 [57.1%]	–
Streptococcus spp.		45 [80.4%]	–
Prevotella spp.		16 [28.6%]	–
Veillonella spp.		11 [19.6%]	–
Rothia spp.		6 [10.7%]	–
Neisseria spp.		5 [8.9%]	–

Data are presented as median (Q1–Q3) or *N* [%].

a Five sites in patients with dentition, three sites in patients without dentition.

*Escherichia coli* and *Streptococci* spp. were identified more frequently in patients admitted from the emergency department than in those transferred from other hospital wards (100% vs. 64.4%, *p* = 0.023 and 100% vs. 72.5%, *p* < 0.001; Figure 3). Moreover, patients in whom no *Streptococci* strains were identified had higher DOT before intubation when compared to those with *Streptococci* strains present (12, IQR 8–26.5 vs. 5, IQR 2–8.5, *p* = 0.013). *Escherichia coli* was more frequently found in patients with diabetes (70% vs. 30%, *p* = 0.025) and CAD (37.5% vs. 10%, *p* = 0.024) than in those without. Finally, non-survivors had lower baseline prevalence of *Lactobacillus* spp. as compared to survivors (48.8% vs. 84.6%, *p* = 0.028).

There were no associations between the sum BOAS scores and microbiological findings, although more detailed analyses revealed *Lactobacillus* spp. positive patients had lower BOAS saliva score as compared to those with no *Lactobacillus* spp. (median 2.5 vs. 3, IQR 2–3 and 2–3 respectively, *p* = 0.045). The BOAS saliva score was lower in patients using antibiotic treatment before intubation (median 2 vs. 3, IQR 2–3 and 2–3 respectively, *p* = 0.05).

The CFU counts were available for samples acquired by the dental brush and from gingival pockets, and the median CFU counts from all sites were 3.0E+5 (6.3E+4–1.0E+6). The median CFU counts of all bacterial strains in dental brushes was 4.0E+5 (1.0E+5–1.4E+6) and in gingival pockets 2.0E+5 (4.0E+4–8.0E+5), with data available for 81.2 and 68.4% samples, respectively. There were no differences in the median CFU counts between the BOAS score categories in dental brushes and gingival pockets (*p* = 0.198). Interestingly, patients with previous antibiotic use had lower CFU counts than those without (median 2.0E+5 [4.0E+4–8.8E+5] vs. 4.5E+5 [1.2E+5–1.5E+6], *p* = 0.007), while patients transferred from the emergency department had higher CFU counts than those transferred from other hospital wards (median 6.0E+5 [2.8E+4–1.5E+6] vs. 3.0E+5 [IQR 4.0E+4–1.0E+6], *p* = 0.016).

**Figure 4:**
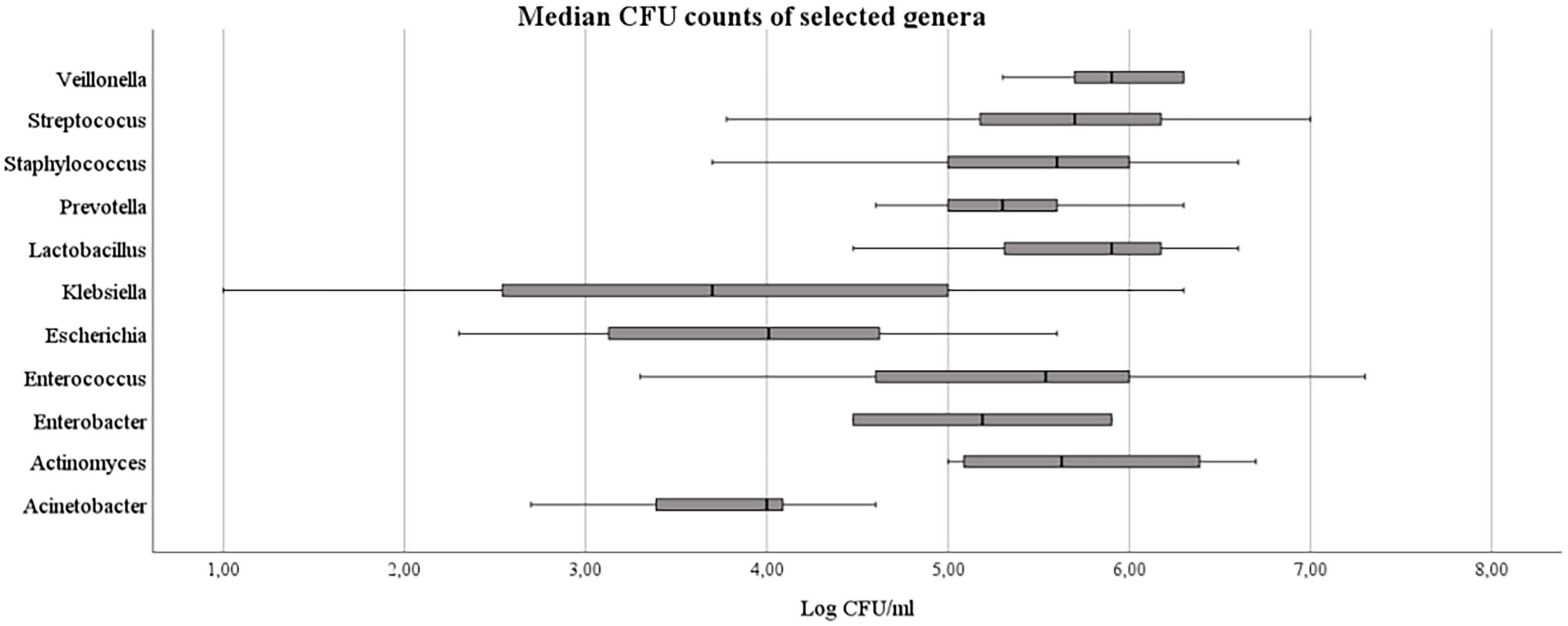
CFU counts of selected identified bacteria genera.

The CFU counts for Gram-positive bacteria were higher than for Gram negative (median 5.0E+5 [1.1E+5–1.5E+6] vs. 1.0E+5 [5.0E+3–3.0E+5], *p* < 0.001). Finally, the highest median CFU counts from all sites were observed for *Enterococcus* spp., *Lactobacillus* spp., *Streptococcus* spp. and *Staphylococcus* spp. (Table 3; Figure 4).

**Table 3:** Baseline quantitative microbiological characteristics of study participants.

	Number of samples with CFU count available	CFU/ml
All bacterial strains	316/881	3.0E+5 (6.2E+4–1.0E+6)
All G+ strains	79	5.0E+5 (1.1E+5–1.5E+6)
All G− strains	237	1.0E+5 (5.0E+3–3.0E+5)
Veillonella spp.	5/11	8.0E+5 (3.4E+5–2.0E+6)
Neisseria spp.	5/15	3.0E+5 (1.5E+5–5.0E+5)
Actinomyces spp.	4/9	6.8E+5 (1.1E+5–4.1E+6)
Prevotella spp.	19/55	2.0E+5 (1.0E+5–4.0E+5)
Streptococcus spp.	11/265	5.0E+5 (1.4E+5–1.5E+6)
Staphylococcus spp.	31/103	4.0E+5 (1.0E+5–1.0E+6)
Lactobacillus spp.	27/87	8.0E+5 (2.0E+5–1.5E+6)
Klebsiella spp.	19/58	5.0E+3 (3.0E+2–1.0E+5)
Escherichia coli	12/38	1.1E+4 (1.2E+3–5.9E+4)
Acinetobacter baumannii	11/50	1.0E+4 (2.0E+3–1.5E+4)
Enterococcus spp.	50/135	3.5E+5 (4.0E+4–1.0E+6)

Data are presented as median (IQR) or *N* [%].

## Discussion

In this report, we comprehensively characterized the oral health condition and cultivable oral bacteriota in COVID-19 patients hospitalized in an ICU with ARDS within 36 h following intubation. In this population, the oral microbiota from mucosal swabs, dental samples, and gingival pockets showed significant qualitative and quantitative dysbiosis and was distinct from healthy patients. SARS-CoV-2 infection, hospitalization in an ICU and mechanical ventilation are important factors leading to oral dysbiosis in patients.

Our study population comprised a homogenous group of patients with COVID-19 infection that were hospitalized in a temporary ICU dedicated to SARS-CoV-2-positive patients. These patients, with severe COVID-19 and ARDS, required specialist medical care, including mechanical ventilation or hemodialysis. Our data demonstrated a significantly higher mortality rate compared to normal COVID admissions, although our election criteria biased patient selection toward the most severe COVID-19 cases. Our study population was also older, with multiple comorbidities that have confirmed deleterious effects on COVID-19 survival, such as diabetes and cardiovascular disease (Harrison et al., 2021).

Our study population presented with moderate or severe dysfunction of oral health. We believe that this resulted from first, poor oral health status in the elderly adult Polish population as presented in one recent report (Malicka et al., 2022). The authors noted that 21% of 70-year-olds were completely toothless. On average, the number of teeth was 12.97 ± 9.5., with 4.7 ± 4.8 teeth in the occlusion. 30.8% of patients wore a partial removable denture, and 25% a complete removable maxillary denture. 20% of study participants had a partial removable denture, and 22.6% had a complete removable mandibular denture. Oral dryness was observed in *ca.* one-third of the studied cohort, nearly 20% had periodontitis and ~30% required treatment for caries. They emphasized that more than 60% of patients required professional dental prophylaxis (Malicka et al., 2022). Secondly, another report from a similar cohort (age and comorbidities) revealed patients hospitalized due to myocardial infarction presented higher plaque and periodontal indices as compared to patients with stable angina pectoris (Wozakowska-Kapłon et al., 2013). Still, this subject is under researched and further exploration is required. Finally, previous studies of ICU COVID-19 patients reported xerostomia, mucosal blistering and ulcers, rash, lip necrosis, and loss of taste and smell (Kamel et al., 2021; Eduardo et al., 2022; Yoshino et al., 2022). These can exacerbate such conditions as periodontitis, being an important risk factor for complications in patients with COVID-19 hospitalized in the ICU (Marouf et al., 2021). Pre-COVID-19 reports showed that hospitalization in an ICU together with mechanical ventilation can have a deleterious effect on oral health (Terezakis et al., 2011), including accumulation of the dental plaque and emergence of mucosal lesions. Due to the deterioration of oral health, critically ill patients in the ICU represent a group vulnerable to further complicates including VAP (Luyt et al., 2020).

Another noteworthy finding was a significant and alarming qualitative and quantitative dysbiosis of the cultivable oral bacteriota in our study population, as early as up to 36 h following intubation. Potentially pathogenic bacteria including *Acinetobacter baumannii, Enterococcus faecalis, Escherichia coli* and *Klebsiella pneumoniae* occurred frequently with large CFU counts in our study population. While previous studies showed that these species are found in the oral cavity, their prevalence was not as high as in our patient population (Beck, 1979; Marshall et al., 2020; Diagnostic testing for SARS-CoV-2 [internet], 2022).

The CFU counts of such species as *Enterococcus faecalis* and *Acinetobacter baumannii* were as high as commensal *Streptococci* and *Staphylococci*, indicating significant abnormalities in the oral bacteriobiota hemeostasis.

In one study, oral rinse samples from COVID-19 patients with a wide spectrum of symptoms showed a comparable extent of oral dysbiosis, with lower bacterial diversity, higher abundance of *Lactobacillus* spp., *Enterococcus* spp., *Acinetobacter baumannii*, and lower amounts of *Gemella* spp., *Fusobacterium* spp. and *Haemophilus* spp. (Soffritti et al., 2021).

The prevalence of *E. coli* was surprisingly high in our population. *Escherichia coli* is not a member of commensal oral microbiota, however it was detected in oral cavities of elderly patients with systemic diseases (Zawadzki et al., 2017). Recently, *E. coli* was reported to successfully colonize a supragingival biofilm (Pérez-Chaparro et al., 2014), so under special nutritional and environmental circumstances, *E. coli* can likely survive and even dominate this niche, especially in immunocompromised patients (Thurnheer and Belibasakis, 2014). Additionally, we found a higher prevalence of *E. coli* in patients admitted from the emergency ward than in those transferred from other hospital wards. To date, there have been no other reports on this issue, warranting further research.

Conversely, *Enterococcus faecalis* was more frequent in patients transferred from other hospital wards than those originating from the emergency department. Previous studies showed similar results, with changes in the oral bacteriobiota composition result from the exposure to hospital bacteria and each subsequent day of hospitalization increases the risk of *Enterococci* infections (Russo Fiorino et al., 2021). Moreover, *E. coli* and *E. faecalis* can employ antagonistic interactions against *S. mutans* (Thurnheer and Belibasakis, 2014), partially explaining our observations.

Our analyses revealed that the prevalence of commensal *Streptococcus* strains was lower in patients with a higher antibiotic consumption prior to intubation. We consider that antibiotics could be a major factor contributing to oral dysbiosis and disappearance of “healthy” commensal strains. Of all antibiotics, β-Lactam antibiotics were most commonly used in our cohort. Prospective cohort studies revealed that Shannon biodiversity index was decreased during amoxicillin treatment and was subject to further reduction in the following 6 months’ time period (Menon et al., 2019; Monroy-Pérez et al., 2020; Nel Van Zyl et al., 2022). The density of Neisseria, Streptococcus and Veillonella strains in the oral cavity was also reported to decrease during treatment with amoxicillin (Larsson Wexell et al., 2016; Moraes et al., 2020). Save commonly used amoxicillin, other groups of antibiotics with various mechanisms of action can influence the oral microbiota and promote the selection of multi-drug resistant strains and their horizontal transmission (Zaura et al., 2015; Moraes et al., 2020). Other factors that can lead to oral dysbiosis include: local and systemic diseases, improper oral hygiene, unbalanced diet, smoking tobacco and immunosuppression (Li et al., 2022).

Lactobacillus was more prevalent in survivors in our study population, but there were no associations with antibiotic use, or with probiotic use according to the care standards in UH wards. One previous study reported the relative abundance of various bacterial genera, including *Lactobacillus* spp. in COVID-19 patients (Soffritti et al., 2021). These findings are notable, as *Lactobacillus* spp. may play some role in the protection against SARS-CoV-2, acting as an inhibitor of viral contamination by multiple mechanisms, including production of metabolites with antiviral activity, stimulation of mucosal immune system cells and local cytokines production (Zrelli et al., 2021).

Previous studies of COVID-19 patients tested saliva or nasopharyngeal swabs (Lloréns-Rico et al., n.d.; Miller et al., 2021). One notable strength of our study is that we investigated mucosal and dental brushes, highly representative for microbiota analysis (Zaura et al., 2001). Considering a proper and complex oral health evaluation, we used the BOAS scale. Among oral assessment tools, BOAS has been proposed as the most appropriate for ICU patients, with the mucosal-dental plaque score most applicable during observation (Ames et al., 2011).

Our study also had some limitations. First, we focused only on the SARS-CoV-2-positive patients. Moreover, in this study we used traditional methods for identification of microorganisms on the species level. As it is known, NGS it also allows obtaining information on non-cultivable microorganisms. But the method we used allowed the establishment of a microbial bank, for future studies in healthcare-associated-infections.

## Conclusion

COVID-19 patients hospitalized in an ICU in the early post-intubation period presented an alarming qualitative and quantitative dysbiosis of the cultivable oral bacteriota. Abnormalities in the oral health status can trigger deterioration and dysbiosis of the oral microbiota. Poor oral hygiene, cough, increased inhalation and mainly mechanical ventilation provide a pathway for oral microorganisms to enter the lower respiratory tract, leading to pneumonia. A proper assessment of oral health can provide information on how to treat and diagnose these patients. Effective oral health care measures are necessary to reduce these infections, especially in severe COVID-19 patients.

## Data availability statement

The raw data supporting the conclusions of this article will be made available by the authors, without undue reservation.

## Ethics statement

The studies involving human participants were reviewed and approved by by the Jagiellonian University Bioethics Committee (Komisja Bioetyczna, Uniwersytet Jagielloński). The patients/participants provided their written informed consent to participate in this study.

## Author contributions

IG-M: conceptualization, investigation, methodology, project administration, resources, supervision, and writing–review and editing. MF: data curation, methodology, investigation, writing–original draft preparation, and writing–review and editing. MK: formal analysis, methodology, visualization, writing–original draft preparation, writing–review and editing. EJ: formal analysis, methodology, validation. DR: formal analysis, methodology, validation. JW-M: methodology, formal analysis, validation, writing–review and editing, resources. All authors contributed to the article and approved the submitted version.

## Funding

This publication was supported by the National Center for Research and Development CRACoV-HHS project (Model of multi-specialist hospital and non-hospital care for patients with SARS-CoV-2 infection) through the initiative “Support for specialist hospitals in fighting the spread of SARS-CoV-2 infection and in treating COVID-19” (contract number SZPITALE-JEDNOIMIENNE/18/2020). The described research was implemented by consortium of the University Hospital in Krakow and the Jagiellonian University Medical College.

## Conflict of interest

The authors declare that the research was conducted in the absence of any commercial or financial relationships that could be construed as a potential conflict of interest.

## Publisher’s note

All claims expressed in this article are solely those of the authors and do not necessarily represent those of their affiliated organizations, or those of the publisher, the editors and the reviewers. Any product that may be evaluated in this article, or claim that may be made by its manufacturer, is not guaranteed or endorsed by the publisher.
