# Supplementary material for: Cultivable oral bacteriota dysbiosis in mechanically ventilated COVID-19 patients
Source: Front Microbiol. 2022 Oct 28;13:1013559. doi: 10.3389/fmicb.2022.1013559 (PMC9651008; doi:10.3389/fmicb.2022.1013559)
Supplement: Supplementary file 1 [file Data_Sheet_1.docx]

**Cultivable oral bacteriota dysbiosis in mechanically ventilated COVID-19 patients**

**Supplementary materials**

| Table S1. WHO clinical progression scale | | |
| --- | --- | --- |
| Patient State | Descriptor | Score |
| Uninfected | Uninfected; no viral RNA detected | 0 |
| Ambulatory mild disease | Asymptomatic; viral RNA detected | 1 |
|  | Symptomatic; independent | 2 |
|  | Symptomatic; assistance needed | 3 |
| Hospitalized: moderate disease | Hospitalized; no oxygen therapy* | 4 |
|  | Hospitalized; oxygen by mask or nasal prongs | 5 |
| Hospitalized: severe diseases | Hospitalized; oxygen by NIV or high flow | 6 |
|  | Intubation and mechanical ventilation, pO_2_/FiO_2_ ≥150 or SpO_2_/FiO_2_ ≥200 | 7 |
|  | Mechanical ventilation pO_2_/FIO_2_ <150 (SpO_2_/FiO_2_) or vasopressors | 8 |
|  | Mechanical ventilation pO_2_/FiO_2_ <150 and vasopressors, dialysis, or ECMO | 9 |
| Dead | Dead | 10 |
| ECMO=extracorporeal membrane oxygenation. FiO2=fraction of inspired oxygen. NIV=non-invasive ventilation. pO_2_=partial pressure of oxygen. SpO_2_=oxygen saturation. *If hospitalized for isolation only, record status as for ambulatory patient.  https://www.who.int/docs/default-source/documents/emergencies/minimalcoreoutcomemeasure.pdf | | |

| Table S2. Beck Oral Assessment Score (BOAS), modified^a^ | | | | |
| --- | --- | --- | --- | --- |
|  | **Score** | | | |
| **Area** | **1** | **2** | **3** | **4** |
| Lips | Smooth, pink, moist, and intact | Slightly dry, red | Dry, swollen isolated blisters | Edematous, inflamed blisters |
|  | | | | |
| Gingiva and oral mucosa | Smooth, pink, moist, and intact | Pale, dry, isolated lesions | Swollen red | Very dry and edematous, inflamed |
|  | | | | |
| Tongue | Smooth, pink, moist, and intact | Dry, prominent papillae | Dry, swollen, tip and papillae are red with lesions | Very dry, edematous, engorged coating |
|  | | | | |
| Teeth | Clean no debris | Minimal debris | Moderate debris | Covered with debris |
|  | | | | |
| Saliva | Thin, watery plentiful | Increase in amount | Scanty and somewhat thicker | Thick and ropy, viscid or mucoid |
|  | | | | |
| Total Score^b^ | 5 No dysfunction | 6–10 Mild dysfunction | 11–15 Moderate dysfunction | 16–20 Severe dysfunction |
|  | | | | |
| Note: Provide moisture more often than oral care | Minimum care every 12 h | Minimum care every 8–12 | Minimum care every 8 h | Minimum care every 4 h |
| - BOAS 0–5 Perform an oral assessment once a day. Follow oral care as outlined in the systematic oral care procedure twice per day. - BOAS 6–10 Perform oral assessments twice a day. Moisten mouth/lips every 4 hours. Follow oral care as outlined in the systemic oral care procedure twice per day. - BOAS 11–15 Perform an oral assessment every shift (every 8–12 h). Follow oral care as outlined in the systematic oral care every shift. Use an ultrasoft toothbrush. Moisten lips and mouth every 2h. - BOAS 16–20 Perform an oral assessment every 4 hours. Follow oral care as outlined. If brushing not possible use soft gauze-wrapped finger. Moisten lips and mouth every 1 – 2 hours. | | | | |
| Modified from Beck and Ames  Ames NJ, Sulima P, Yates JM, McCullagh L, Gollins SL, Soeken K, Wallen GR. Effects of systematic oral care in critically ill patients: a multicenter study. Am J Crit Care. 2011 Sep;20(5):e103-14. doi: 10.4037/ajcc2011359. | | | | |

| Table S3. Comparison of participants according to survival status. | | | | |
| --- | --- | --- | --- | --- |
| Characteristics | Data available N=56 | Survivors  N=13 | Non-survivors  N=43 | P value |
| Characteristics |  |  |  |  |
| Age [years] | 56 | 59.8 (17.7)  65 (43.5-74) | 68.5 (10.2)  67 (62-76) | 0.165 |
| Female [n (%)] | 56 | 7 (53.8%) | 17 (39.55) | 0.524 |
| BMI [kg/m^2^]* | 35 | 34.5 (2.9)  34.9 (31.7-36.2) | 31.4 (6.1)  31.2 (25.8-38.4) | 0.202 |
| WHO ordinal scale, on admission to an ICU | 56 | 7 (6-8) | 6 (5-9) | 0.381 |
| Source of admission [n (%)] | 56 |  |  | 0.841 |
| Emergency ward |  | 4 (30.8%) | 12 (27.9%) |  |
| Hospital ward |  | 9 (69.2%)) | 31 (72.1%) |  |
| Time from COVID-19 diagnosis* to intubation [days] | 53 | 5.6 (5.9)  5.5 (0.3-8.5) | 7.4 (6.8)  7 (1-12) | 0.410 |
|  |  |  |  |  |
| Baseline BOAS, sum score | 49 | 11.2 (2.3)  11 (10.3-12.8) | 11.8 (3.0)  12 (10-14) | 0.371 |
|  |  |  |  |  |
| Laboratory findings |  |  |  |  |
| CRP, first recorded [mg/l] | 55 | 140.1 (72.3)  161.5 (76.5-204.3) | 163.5 (105.2)  167 (72-238) | 0.501 |
| PCT, first recorded [ng/ml] | 55 | 10.6 (29.0)  0.3 (0.06-1.06) | 3.5 (15.3)  0.4 (0.16-0.87) | 0.386 |
| IL-6, first recorded [pg/ml] | 54 | 93.7 (93.7)  67.2 (20.5-135.6) | 154.7 (258.7)  69.3 (28.9-144.9) | 0.677 |
| WBC, first recorded [10^3^/mm^3^] | 54 | 9.9 (6.4)  9.5 (3.97-14.9) | 11.1 (7.9)  8.9 (5.3-14.0) | 0.839 |
| HbA1c [%] | 27 | 5.5 (0.3)  5.4 (5.2-NA) | 7.2 (1.6)  6.6 (6.3-7.9) | 0.008 |
|  |  |  |  |  |
| Comorbidities [n (%)] |  |  |  |  |
| COPD | 56 | 0 | 3 (7.0%) | 1 |
| Smoking | 52 |  |  | 0.306 |
| Active smoking |  | 0 | 5 (12.5%) | NA |
| History of smoking |  | 1 (8.3%) | 1 (2.5%) |  |
| Diabetes | 56 | 2 (15.4%) | 18 (41.9%) | 0.106 |
| Neoplasm | 56 |  |  | 0.704 |
| In treatment |  | 1 (7.7%) | 3 (7.0%) |  |
| <5 years ago |  | 0 | 4 (9.3%) |  |
| >5 years ago |  | 0 | 3 (7.0%) |  |
| Hypertension | 56 | 5 (38.5%) | 21 (48.8%) | 0.545 |
| Coronary artery disease | 56 | 4 (30.8%) | 12 (27.9%) | 1 |
| Heart failure | 56 | 1 (7.7%) | 5 (11.6%) | 1 |
| CKD | 56 | 2 (15.4%) | 2 (15.4%) | 0.227 |
|  |  |  |  |  |
| Pharmacotherapy before hospitalization [n (%)] | 51 |  |  |  |
| ACEI/ARB |  | 4 (33.3%) | 17 (43.6%) | 0.739 |
| Beta blocker |  | 2 (16.7%) | 16 (41.0%) | 0.174 |
| CCB |  | 2 (16.7%) | 9 (23.1%) | 1 |
| Diuretic |  | 2 (16.7%) | 10 (25.6%) | 0.706 |
| Statin |  | 1 (8.3%) | 10 (25.6%) | 0.422 |
| Aspirin |  | 0 | 7 (17.9%) | 0.177 |
| VKA/NOAC |  | 3 (25.0%) | 3 (5.9%) |  |
| Metformin |  | 0 | 9 (23.1%) | 0.094 |
| Insulin |  | 2 (16.6%) | 6 (15.4%) | 1 |
| Systemic steroid therapy |  | 1 (8.3%) | 4 (10.3%) | 1 |
| Immune suppressive therapy |  | 2 (16.7%) | 2 (5.1%) | 0.232 |
|  |  |  |  |  |
| In-hospital pharmacotherapy before intubation [n (%)] | 52 |  |  |  |
| Steroid therapy |  | 7 (58.3%) | 33 (82.5%) | 0.119 |
| Remdesivir |  | 2 (16.7%) | 13 (33.3%) | 0.470 |
| Tocilizumab |  | 1 (8.3%) | 10 (25.0%) | 0.421 |
| Antibiotic |  | 7 (58.3%) | 26 (65.0%) | 0.739 |
| DOT before intubation [days] | 33 | 17 (17.9)  13.5 (6.25-23.5) | 7.9 (6.1)  6 (4-11) | 0.733 |
| Anti-fungal agents | 52 | 3 (25.0%) | 3 (7.5%) | 0.127 |
| PPI | 52 | 6 (50.0%) | 18 (45.0%) | 1 |
| *defined as first positive SARS-CoV-2 nasopharyngeal swab  data are presented mean (SD), median (Q1-Q3) or N [%]; BMI – body mass index; WHO – World Health Organization; ICU – intensive care unit; UH – University Hospital Cracow, Poland; CRP – C-reactive protein; PCT – procalcitonin; IL-6 – interleukin 6, WBC – white blood cell count; COPD – chronic obstructive pulmonary disease; CKD – chronic kidney disease; ACEI - angiotensin-converting-enzyme inhibitors; ARB - angiotensin II receptor blockers; CCB – calcium channel blockers; VKA – vitamin K antagonists; NOAC – new oral anticoagulant; PPI – proton pump inhibitor; BOAS - Beck Oral Assessment Scale, DOT – days of antibiotic therapy. | | | | |

| Table S4. List of all identified species. | | |
| --- | --- | --- |
| Species | Number of patients with strain identified [N (%)] | |
| Acinetobacter baumannii | 13 | 23.2% |
| Acinetobacter spp | 1 | 1.8% |
| Actinomyces graevenitzii | 2 | 3.6% |
| Actinomyces odontolyticus | 3 | 5.4% |
| Atopobium parvulum | 2 | 3.6% |
| Bacillus idriensis | 1 | 1.8% |
| Bifidobacteriuim spp | 2 | 3.6% |
| Capnocytophaga sputigena | 2 | 3.6% |
| Corynebacterium amycolatum | 1 | 1.8% |
| Corynebacterium striatum | 1 | 1.8% |
| Enterobacter cloacae | 1 | 1.8% |
| Enterococcus avium | 1 | 1.8% |
| Enterococcus faecalis | 22 | 39.3% |
| Enterococcus faecium | 14 | 25.0% |
| Enterococcus gallinarum | 1 | 1.8% |
| Escherichia coli | 10 | 17.9% |
| Fusobacterium nucleatum | 1 | 1.8% |
| Gemella haemolysans | 1 | 1.8% |
| Granulicatella adiacens | 1 | 1.8% |
| Hafnia alvei | 1 | 1.8% |
| Klebsiella oxytoca | 4 | 7.1% |
| Klebsiella pneumoniae | 11 | 19.6% |
| Klebsiella variicola | 1 | 1.8% |
| Lactobacillus crispatus | 3 | 5.4% |
| Lactobacillus fermentum | 13 | 23.2% |
| Lactobacillus salivarius | 2 | 3.6% |
| Lactobacillus spp | 26 | 46.4% |
| Leuconostoc pseudomesenteroides | 1 | 1.8% |
| Morganella morganii | 2 | 3.6% |
| Neisseria mucosa | 1 | 1.8% |
| Neisseria spp | 5 | 8.9% |
| Obesumbacterium proteus | 1 | 1.8% |
| Pediococcus pentosaceus | 1 | 1.8% |
| Pediococcus spp | 1 | 1.8% |
| Peptostreptococcus anaerobius | 1 | 1.8% |
| Prevotella denticola | 3 | 5.4% |
| Prevotella melaninogenica | 11 | 19.6% |
| Prevotella nigrescens | 1 | 1.8% |
| Prevotella salviae | 3 | 5.4% |
| Prevotella veroralis | 1 | 1.8% |
| Proteus mirabilis | 3 | 5.4% |
| Pseudomonas aeruginosa | 2 | 3.6% |
| Raoultella ornithinolytica | 1 | 1.8% |
| Rothia mucilaginosa | 6 | 10.7% |
| Serratia marcescens | 1 | 1.8% |
| Staphylococcus aureus | 9 | 16.1% |
| Staphylococcus capitis | 1 | 1.8% |
| Staphylococcus epidermidis | 16 | 28.6% |
| Staphylococcus haemolyticus | 13 | 23.2% |
| Staphylococcus hominis | 2 | 3.6% |
| Stenotrophomonas maltophilia | 1 | 1.8% |
| Streptococcus agalactiae | 2 | 3.6% |
| Streptococcus anginosus | 13 | 23.2% |
| Streptococcus constellatus | 2 | 3.6% |
| Streptococcus cristatus | 3 | 5.4% |
| Streptococcus gordonii | 6 | 10.7% |
| Streptococcus intermedius | 1 | 1.8% |
| Streptococcus mitis | 1 | 1.8% |
| Streptococcus mutans | 1 | 1.8% |
| Streptococcus parasanguinis | 20 | 35.7% |
| Streptococcus pneumoniae | 5 | 8.9% |
| Streptococcus salivarius | 2 | 3.6% |
| Streptococcus sciuri | 1 | 1.8% |
| Streptococcus spp | 39 | 69.6% |
| Veillonella dispar | 5 | 8.9% |
| Veillnella parvula | 2 | 3.6% |
| Veillonella atypica | 2 | 3.6% |
